# Understanding risk communication for prevention and control of vector-borne diseases: A mixed-method study in Curaçao

**DOI:** 10.1371/journal.pntd.0008136

**Published:** 2020-04-13

**Authors:** Vaitiare Mulderij-Jansen, Jelte Elsinga, Izzy Gerstenbluth, Ashley Duits, Adriana Tami, Ajay Bailey

**Affiliations:** 1 University of Groningen, University Medical Center Groningen, Department of Medical Microbiology and Infection Prevention, Groningen, The Netherlands; 2 International Development Studies, Department of Human Geography and Spatial Planning, Faculty of Geosciences, Utrecht University, Utrecht, The Netherlands; 3 Curaçao Biomedical & Health Research Institute, Department of Epidemiology, Willemstad, Curaçao; 4 Epidemiology and Research Unit, Ministry of Health Environment and Nature of Curaçao, Willemstad, Curaçao; 5 Red Cross Blood Bank Foundation, Willemstad, Curaҫao; 6 Curaçao Biomedical & Health Research Institute, Department of Immunology, Willemstad, Curaçao; 7 Transdisciplinary Centre for Qualitative Methods, Manipal Academy of Higher Education, Manipal, India; Goethe University, GERMANY

## Abstract

**Background:**

Risk communication (RC) is an essential tool for the prevention and control of diseases as it impacts risk perception, increases awareness and might change behaviour. It is the interactive exchange of information about risks among experts and people. Effective RC can minimize the impact that diseases such as dengue, chikungunya and Zika have on populations. This study aimed to understand RC regarding vector-borne diseases in its social context and from the viewpoint of the audience to strengthen RC strategies in Curaçao.

**Methods:**

In 2015, a cross-sectional mixed-method study applying focus group discussions (n = 7), in-depth interviews (n = 20) and a structured survey questionnaire (n = 339) was done in Curaçao. The study was designed based on the Health Belief Model and the Theory of Planned Behaviour. In addition, the Social Amplification of Risk Framework and the theory of cultural schemas were applied to understand RC in the social context.

**Results:**

Television, radio and newspapers were the most important channels of information regarding dengue and chikungunya. Moreover, individuals also reported receiving information via social media, the internet and family/friends. Interestingly, the use of internet to obtain information diminished with age, while females were more likely to use internet compared to men. These key findings were statistically significant. An important outcome was that the risk perception towards chikungunya at the beginning of the outbreak was attenuated. This might be due to the (perceived) lack of RC before the epidemic. This same risk perception was amplified later during the outbreak by the increased exposure to information. Lastly, we show how cultural schemas influence people’s perception regarding preventive measures and treatment of chikungunya and dengue.

**Conclusions:**

Data obtained emphasise the importance of understanding the *user* of media platforms and sharing information in a timely fashion through a transparent process with the content that convinces people of the seriousness of the matter.

## Author summary

Vector-borne diseases (VBDs) such as dengue, chikungunya and Zika are an increasing public health concern worldwide. The mentioned VBDs are transmitted to humans through the bite of an infected mosquito from the *Aedes* species. Preventing or reducing VBDs continues to depend mainly on vector control and interrupting human-vector contact. Risk communication (RC) is the interactive exchange of information about hazards among experts and individuals. As it influences individuals’ behaviour, a better understanding of how it works is vital to improving RC strategies in the context of VBD prevention and control strategies. Our study highlighted the complexity of this matter as we found that there are multiple factors, including the volume of information, trust, experience with a similar disease and cultural schemas that determine how people cope with risk and information. We recommend broadening the use and scope of media platforms to share information and to customise the messages taking the cultural schemas of the community into account.

## Introduction

Dengue, chikungunya, and Zika are an increasing global public health concern as a result of their dramatically increased burden of disease and rapid geographical spread [1–4]. Dengue is the most important arthropod-borne viral disease in humans. It is endemic in 125 countries mostly in America, South-East Asia and Western Pacific regions [5]. Approximately 4 billion people are now at risk of dengue [2]. A dengue virus (DENV) infection causes a flu-like illness and occasionally progresses to severe forms of dengue: dengue hemorrhagic fever (DHF) and dengue shock syndrome (DSS) [5]. Individuals infected with chikungunya virus (CHIKV) generally have symptoms such as high fever, skin rash and debilitating polyarthralgia, which usually persist 1–2 weeks. However, a proportion of cases progresses to the chronic stage of CHIKV infection, in which some symptoms, such as arthralgia, last for months to years [6, 7]. Zika virus (ZIKV) infection has been linked to adverse fetal outcomes, including microcephaly and other congenital abnormalities in the developing fetus and newborn. Also, it has been associated with Guillain- Barré syndrome (GBS)[3], but one must take into account that GBS is also associated with other bacterial and viral infections, e.g. Campylobacter jejuni, Influenza and other Vector-Borne Diseases (VBDs) such as dengue and chikungunya [8–10]. These VBDs present with flu-like symptoms and are generally self-limiting, barring the development of the aforementioned complications [11].

In the Caribbean, the primary vector of DENV (Flavivirus), CHIKV (Alphavirus) and ZIKV (Flavivirus) is *Aedes aegypti* [12]. *Ae*. *aegypti* is a tropical and subtropical mosquito species widely distributed around the world, mostly between latitudes 35 °N and 35 °S [13]. Given the vector distribution, countries located in the tropical and subtropical regions are susceptible to its invasion and spread [13]. In Curaçao, a Caribbean island within the Kingdom of the Netherlands, all four serotypes of dengue (DENV 1–4) have circulated during the past two decades [14]. According to local health authorities, dengue epidemics occur cyclically. CHIKV caused a significant outbreak in 2014–2015, affecting up to 50% of the population (Ministry of Health, Environment and Nature of Curaçao, 2016). In 2016, the introduction of ZIKV caused another major outbreak in the country. The Department of Epidemiology and Research of the Ministry of Health (MoH) reported that since December 2015 up to August 2017, a total of 2314 laboratory-confirmed ZIKV cases were recorded. However, it should be noted that confirmed cases only represent a fraction of those who were infected, as up to 80% of the individuals with ZIKV infection are asymptomatic and not everyone experiencing symptoms, which may be very mild, will visit a health facility [15]. The MoH estimates that at least 50% of the local population had been infected with ZIKV (I. Gerstenbluth, personal communication). This is comparable to the reported attack rates in French Polynesia (66%) and Yap Island (73%) [16, 17].

The treatment of dengue, chikungunya, and Zika relies on supportive treatment and symptom relief since there is no antiviral treatment available. Considering the possible complications of these viral diseases, prevention, prompt diagnosis and adequate intervention at the onset of disease symptoms are the main options for reducing the burden of disease. To date, no vaccines are available for Zika, and chikungunya [3, 18]. There is a licensed dengue vaccine available. However, it is not suitable for large-scale use [19]. Thus, the prevention or reduction of the transmission of the mentioned VBDs hinges primarily on control of the mosquito vectors *(e*.*g*. *environmental management*, *biological and chemical control)* and interruption of human-vector contact *(e*.*g*. *individual protection including the use of repellents*, *clothing that minimises skin exposure*, *and household protection including window and door screens*, *and air-conditioning)*[13, 20].

Risk communication (RC) refers to the exchange of real-time information, including advice and opinions between experts and individuals facing threats to their health, economic or social well-being [21]. It is an essential tool for the prevention and control of diseases because it can supply individuals with the knowledge needed for an optimal decision-making process [22, 23]. The Health Belief Model (HBM) is one of the most widely used and validated theoretical models to understand the decision-making process and the health-seeking behaviour of individuals [24]. Core concepts in the HBM are the perceived susceptibility and the perceived severity of the condition, leading to the perceived threat. The perception of threat is integral in the decision-making processes of individuals because it plays an essential role in motivating health behaviour change [25]. An essential concept in the HBM that influences the perceived risk and health-seeking behaviour is called “cues to action”. Cues to action refer to strategies that activate readiness. According to a recent study published by Metta et al., individuals’ previous experiences in managing conditions similar to malaria were considered to be cues to action, because their past experiences seem to have informed their self-medication decisions in the context of malaria [26]. According to Hochbaum (1958), cues to action could be cultural schemas of individuals or information that individuals receive from media, informal and formal networks [27]. Cultural schemas are deeply internalised and largely unconscious networks of associations built up over time that facilitate perception, interpretation, and action [28]. Thus, cultural schemas are frameworks of a specific culture that exist in an individual’s thoughts and have the ability to instigate actions. The cultural schemas are also shaped by the political context, which then influences the relation between the State and the individual/community[29].

Risk perception is also essential for RC because it determines which hazards individuals care about and how they deal with them. RC models such as risk perception, mental noise, negative dominance, and trust determination have been developed to understand how individuals perceive risk, how they process risk information, and how they make decisions about them [22]. However, recent research indicates a general shift in focus towards models emphasising the importance of social-cultural factors for public acceptance of risk messages [30]. The Social Amplification of Risk Framework (SARF), developed by Kasperson et al. has been prominent on research agendas in trying to understand the gaps between risk perception research and the social context [31]. The framework seeks to explain how information processes, institutional structures, the behaviour of social groups, and individual responses shape the social experience of risk [32, 33]. The SARF states that risk events interact with individual psychological, social and cultural factors in ways that risks are amplified, receiving public attention, or attenuated, receiving less public attention [32].

As essential as it is, RC in the context of vector/mosquito-borne disease remains a neglected topic in the scientific literature [22, 34–38]. Although the mentioned studies give useful insights, they fail to explain RC through the individual point of view and in the social context. Therefore in this paper, we aim to understand RC regarding VBDs in the social context and from the audience’s point of view. The audience in this study refers to people that experienced VBDs and that live in Curaҫao. It is essential to take the audience’s perception into account when studying RC because it permits RC experts to understand the audience’s expectations, opinions and beliefs. The aim of this study will be addressed by seeking to understand (I) the user of the channels of information, (II) the risk perception regarding dengue and chikungunya, (III) the influence of cultural schemas on information, perceptions and preferences, and finally (IV) the information channels that individuals consider trustworthy. This knowledge will lead to applicable community-focused health messages and strengthen the preparedness and performance of RC for future epidemics in Curaçao, and other countries vulnerable to VBDs. We will use a mixed-methods approach based on the SARF, HBM, and the cultural schemas theory to investigate RC regarding VBDs.

## Methods

### Study design

In June and July 2015, a cross-sectional mixed-method (quantitative and qualitative) study using focus group discussions (FGDs), in-depth interviews (IDIs), and individual questionnaires (survey), was performed to assess community participation in Mosquito Breeding Site Control (MBSC) in Curaҫao [39]. The study was designed based on an integrated theoretical framework of the HBM and Theory of Planned Behaviour (TPB). This current study used the SARF, HBM and the theory of cultural schemas, as an analytical interpretive framework, to understand RC among the recruited study participants of the aforementioned cross-sectional mixed-method study. Three concepts of the HBM were incorporated in the SARF. The SARF was applied to explore which channels of information inform individuals about VBDs and the concepts of perceived susceptibility and perceived severity of the HBM were applied to understand the risk perception of the study population. The information coming from different channels was considered as a cue to action. The theory of cultural schemas was also incorporated in our integrated conceptual framework to understand the cultural dimensions in the processing of information at the individual level. Both SARF and cultural schemas were not applied at the time of data collection but were used for analysis and result interpretation. In this study, the qualitative data were analysed first and were validated and supported through quantitative analyses.

### Study site

Curaçao (capital Willemstad) is an island in the southern Caribbean Sea, located ±65 km north of the Venezuelan coast. It has a surface area of 444 km^2^. In October 2010, Curaçao became an autonomous country within the Kingdom of the Netherlands. The government of Curaçao is established under the framework of a parliamentary democracy, with a Prime Minister functioning as the head of government [40]. According to the Central Bureau of Statistics (CBS) of Curaçao, the estimated population was approximately 160,337 inhabitants, with an average population density of 361/km^2^ on January 1st, 2017 [41]. There are different ethnic backgrounds, with an Afro-Caribbean majority and minorities such as Dutch, French, Latin American, South- and East- Asian, Portuguese and Levantine people [42]. Official languages of Curaçao are Papiamentu, Dutch and English. However, Spanish is also widely spoken. The majority of the population of Curaçao is concentrated around the main economic areas of the island, the south-central part, where the oil refinery, the harbour, the government, and virtually all large employers are located [43].

### Qualitative methods

#### Study population

Seven representative population groups of Curaçao were selected to participate in FGDs (n = 50 participants)[39]. The following groups were included; (I) residents born in the Netherlands; (II) local youth; (III) interviewers of the survey; (IV-VII) individuals from the neighbourhoods of Rooi Santu, Seru Fortuna, Souax and Koraalspecht)(S1 Table). The neighbourhoods Rooi Santu, Seru Fortuna, Souax and Koraalspecht comprise individuals of all socio-economic statuses. FGDs with the interviewers of the survey and residents born in the Netherlands were used as comparison groups. An FGD with the local youth was conducted to compare the experiences and perceptions of the local youth with adults. The IDIs were conducted among twenty adults with laboratory-confirmed CHIKV infection. The number of FGDs and IDIs needed in the study was determined after data saturation had been reached [44]. Study subjects for qualitative research were recruited using the snowball recruitment technique, key informants, and via community centres [39].

#### Data collection

The FGDs consisted of 4–10 individuals per group with a similar socio-economic background. The FGD was conducted in Dutch or Papiamentu, depending on the preferences of the participants [39]. IDIs and FGDs were recorded in Dutch and Papiamentu, translated to Dutch, transcribed and analysed using codes and code families.

#### Data analysis

Qualitative data were analysed using several codes, which refer to an idea, issue, topic or opinion that is evident in the data [45]. Some of the codes were raised by the study participants themselves (inductive) while others were prompted by the interviewers using topics in the interview guide that were derived from literature and existing theories (deductive). We employed two cycles of inductive and deductive coding. In the first cycle, 59 codes were used when analysing the FGDs and IDIs. The coding list of both the FGDs and IDIs can be found in the supporting file S2 Table. These codes were assigned to 9 code families, which were analysed in the second cycle of analysis. The following code families were identified; (I) channel of information, (II) the user of the channel of information, (III) preferred channels of information, (IV) risk perception, (V) transmission routes, (VI) symptoms, (VII) preventive measures, (VIII) treatment options, (IX) trust in the channel of information. The qualitative data were analysed using Atlas.ti (version 8.2.0).

### Quantitative methods

#### Study population

During the 2014–2015 chikungunya epidemic, adult subjects with serologically or clinically confirmed CHIKV infection were selected from a representative patient sample from 20 general practitioners (GP) across the island [39]. Two of the subjects were self-diagnosed. Eligible subjects were either contacted by phone or visited at their residence for inclusion. Subjects consenting to participate in the study were interviewed at their homes. A total of 339 individuals joined the study (response rate: 82,5%). The characteristics of the study participants of the survey are presented in S3 Table.

#### Data collection

A questionnaire containing pre-coded and open questions was designed in Dutch and piloted, adapted and translated into English, Papiamentu, and Spanish. Local, experienced interviewers working for the CBS of Curaçao and speaking the above mentioned four languages conducted the interviews. The questionnaire addressed socio-demographic characteristics and chikungunya chronic disease persistence by applying the Curaçao Long-term Chikungunya Sequelae Score (CLTCS Score) to measure the perceived severity of long-term chikungunya disease [46]. The concepts of the HBM *(e*.*g*., *perceived susceptibility*, *perceived severity and cues to action)*, aspects of risk communication *(e*.*g*., *channels of information)*, transmission routes and treatment options of dengue and chikungunya infection were measured using multiple 5-point Likert-items *(e*.*g*. *1*: *do not agree at all to 5*: *fully agree)* or binary items *(e*.*g*. *“yes or no”)*.

#### Data analysis

SPSS Data Entry Station (SPSS Inc. 1996–2003, version 4.0.0) was used for quantitative data entry. All quantitative data were analysed by IBM SPSS Statistics for Windows, Version 25.0. Armonk, NY: IBM Corp. Associations between categorical variables were analysed using the Chi-square test, the Fisher’s exact test or the Fisher-Freeman-Halton exact test when appropriate. Cramér’s V and Phi coefficient were used to measure the strength of association between two nominal variables [47, 48]. Multicollinearity was assessed using the variance inflation factor (VIF). In this study, no collinearity was found. Univariate and multivariate logistic regression models were computed to examine the association between channels of information and socio-demographic characteristics. Characteristics that were significantly associated with the dependent variable, at a 0.10 alpha level in univariate analyses, were included in the multivariate regression model [49]. The Enter method was used. For all other analyses, significance was determined at an alpha level of 0.05.

### Ethics statement

The study was approved by the Medical Ethical Board of the Sint Elisabeth Hospital Curaçao (METC SEHOS; reference number: 2015–002). All participants signed a written informed consent. The data was anonymised and stored in files accessible only to the principal investigators.

## Results

The results are presented here by the following key themes; the user of the channels of information, the risk perception towards dengue and chikungunya, the influence of cultural schemas on information, perceptions and preferences and trust in the channels of information. The characteristics of the participants of the FGDs and IDIs, and the survey are presented in the supporting file: S1 and S3 Tables.

### The user of the channels of information

The majority of the participants of both FGDs and IDIs reported having received information regarding dengue and chikungunya mainly via television, radio, and newspapers. A close analysis of the qualitative data revealed that the interpersonal channels of information *(e*.*g*. *friends*, *family members and colleagues who were infected with CHIKV*, *conventional and alternative medicine practitioners)* had also played an essential role in the provision of information regarding these diseases. These results are in line with those obtained in the cross-sectional study and published previously [39]. This paper went a step further in understanding RC by analysing the associations between channels of information *(e*.*g*. *television*, *radio*, *newspaper*, *internet sites*, *social media*, *GP and family/friends)* and socio-demographic characteristics *(e*.*g*. *age*, *gender*, *education*, *income and occupation)* of the participants of the mentioned cross-sectional study.

Univariate analyses showed that the younger participants *(18–50 years n = 163)* were more likely to receive information regarding chikunguya via social media (18.4% vs 5.7%, *p* <0.001), internet (24.5% vs 6.9%, *p* <0.001) and family/friends (49.7% vs 35.1%, *p* = 0.01) compared to the older participants *(≥ 51 years n = 174)*(S4 Table). In the context of dengue, the younger participants *(18–50 years n = 161)* were more likely to receive information via their GPs (22.4% vs 10,1%, *p* = 0.003), social media (13.7% vs 3.6%, *p* <0.001), and internet (19.3% vs 6.5%, *p* <0.001) compared to the older participants *(≥ 51 years n = 168)*(Table 1). Futhermore, women *(n = 246)* were more likely to receive information regarding chikunguya from the internet (18.3% vs 7.7%, *p* = 0.02) and family/friends (46.7% vs 29.7%, *p* <0.001) compared to men *(n = 91)*(S4 Table).

**Table 1:** Selected comparisons between socio-demographic characteristics and the use of channels of information regarding dengue.

**Age vs GPs**	**Users n (%)**	**Total number of subjects**	***p-*value**
18–50 years	36 (22.4)	161	0.003^^^
≥ 51 years	17 (10.1)	168	
**Age vs Social media**	**Users n (%)**	**Total number of subjects**	***p-*value**
18–50 years	22 (13.7)	161	< 0.001^^^
≥ 51 years	6 (3.6)	168	
**Age vs Internet**	**Users n (%)**	**Total number of subjects**	***p-*value**
18–50 years	31 (19.3)	161	< 0.001^^^
≥ 51 years	11 (6.5)	168	
**Education vs Internet**	**Users n (%)**	**Total number of subjects**	***p-*value**
Illiterate and primary school	3 (4.0)	75	< 0.001^^^
Secondary school	13 (10.6)	123	
Intermediate vocational school	13 (15.5)	84	
Higher vocational education	13 (27.7)	47	
**Education vs GPs**	**Users n (%)**	**Total number of subjects**	***p-*value**
Illiterate and primary school	9 (12.0)	75	0.01^^^
Secondary school	26 (21.1)	123	
Intermediate vocational school	6 (7.1)	84	
Higher vocational education	12 (25.5)	47	
**Education vs Newspapers**	**Users n (%)**	**Total number of subjects**	***p-*value**
Illiterate and primary school	26 (34.7)	75	0.01^^^
Secondary school	73 (59.3)	123	
Intermediate vocational school	42 (50.0)	84	
Higher vocational education	27 (57.4)	47	
**Occupation vs GPs**	**Users n (%)**	**Total number of subjects**	***p-*value**
Unemployed	5 (8.2)	61	0.01^^^
Paid job (manual)	24 (17.3)	139	
Paid job (not manual)	18 (26.9)	67	
Retired	6 (9.7)	62	
**Income** *(ANG/month)*^1^ **vs Television**	**Users n (%)**	**Total number of subjects**	***p-*value**
0–999	27 (81.8)	33	0.04^^^
1000–2499	101 (75.4)	134	
2500–4999	86 (74.1)	116	
≥5000	24 (55.8)	43	

^1^Antillean Guilders, 1 ANG = 0.54 USA dollars and 0.47 EUR.

^^^ Chi-square test

We also found that the tendency to use the internet for information on dengue increased with the level of education (Table 1). The same trend was observed in the usage of internet and social media to seek information regarding chikungunya (S4 Table). This trend also holds true with respect to the usage of GPs as a source of information on dengue (Table 1). We also found a significant association between the usage of newspapers as a channel of information regarding dengue and level of education (Table 1).

With regards to employment, we found that the usage of internet and social media as channels of information on chikungunya was higher in individuals that had a paid job compared to individuals who were unemployed or retired (S4 Table). Individuals with paid jobs were more likely to receive information regarding dengue from their GPs compared to individuals who were unemployed or retired (Table 1). Univariate analysis also showed that the usage of television as a channel of information regarding dengue diminished with increasing income (Table 1). Although not significant, the use of internet and social media showed an increasing trend with income (S4 Table). Only the significant associations are presented in Table 1.

Most of the socio-demographic characteristics used in this study were associated with both social media and internet usage to seek information regarding chikungunya. Therefore, logistic regression was performed to understand these associations further. The use of the internet and social media are strongly correlated *(Phi Coefficient and Cramer’s V test*: *0*.*48*, *p = 0*.*00)* [48]. Therefore, we focus only on the association between the use of the internet and socio-demographic characteristics.

Univariate analysis revealed a negative association between age and the use of the internet, while female gender was positively associated with internet usage. The use of internet increased with the level of education. Individuals having a paid (non-manual) job were 2.72 times more likely to use the internet compared with unemployed individuals. Furthermore, individuals earning ≥5000 guilders per month were 3.23 times more likely to use the internet compared to individuals who earned ≤999 guilders per month (Table 2). After adjusting for education, occupation and income, age and gender were still significantly associated with the use of the internet (Table 3). The data of this study showed that as age increases the use of the internet decreases 5% per each year of age. Also, female subjects were 3.05 times more likely to use the internet compared to male subjects.

**Table 2:** Univariate analysis of socio-demographic characteristics associated with the use of the internet to seek information regarding chikungunya.

Variable	Use of the internet			95% CI for exp b	*P-*value*
	Users n (%)	Total number of subjects	Crude OR		
**Age** *(years)*^3^	52 (15.4)	337	0.95	0.93–0.97	< 0.001*
**Gender**^3^					
Male	7 (7.7)	91	1	-	-
Female	45 (18.3)	246	2.69	1.16–6.20	0.02*
**Education**^3^					0.02*
Illiterate and primary school	4 (5.0)	80	1	-	-
Secondary school	21 (16.5)	127	3.76	1.24–11.41	0.02*
Intermediate vocational school	15 (18.1)	83	4.19	1.33–13.24	0.01*
Higher vocational education	12 (25.5)	47	6.51	1.96–21.63	< 0.001*
**Occupation**^1^^,^^3^					0.01*
Unemployed	7 (11.1)	63	1	-	-
Paid job (manual)	25 (17.5)	143	1.69	0.69–4.15	0.25
Paid job (not manual)	17 (25.4)	67	2.72	1.04–7.10	0.04*
Retired	3 (4.7)	64	0.39	0.10–1.60	0.19
**Income** *(ANG/month)*^2^^,^^4^					0.17
0–999	3 (8.6)	35	1	-	-
1000–2499	16 (11.9)	135	1.43	0.39–5.23	0.58
2500–4999	21 (17.8)	118	2.31	0.65–8.26	0.20
≥5000	10 (23.3)	43	3.23	0.81–12.83	0.09*

^1^The variable unemployed includes student, housewife and volunteer.

^2^Antillean Guilders, 1 ANG = 0.54 USA dollars and 0.47 EUR.

^3^For two participants the data regarding internet usage was missing. These two participants were excluded from the data analysis (n = 337).

^4^For eight participants the data regarding internet usage was missing. These eight participants were excluded from the data analysis (n = 331).

*Significance was determined at an alpha level of 0.10.

**Table 3:** Multivariate analysis of socio-demographic characteristics associated with the use of the internet to seek information regarding chikungunya.

Variable	Use of the Internet		*p-*value*
	OR	95% CI for exp b	
**Age** *(years)*	0.95	0.93–0.98	< 0.001*
**Gender** *(Female)*	3.05	1.21–7.67	0.02*
**Education**			0.76
Illiterate and primary school	1	-	-
Secondary school	1.70	0.51–5.73	0.39
Intermediate vocational school	1.83	0.50–6.61	0.36
Higher vocational education	2.15	0.53–8.70	0.28
**Occupation**^1^			0.60
Unemployed	1	-	-
Paid job (manual)	2.00	0.73–5.45	0.18
Paid job (not manual)	1.84	0.59–5.76	0.29
Retired	1.64	0.30–8.94	0.56
**Income** *(ANG/month)*^2^			0.97
0–999	1	-	-
1000–2499	0.93	0.24–3.64	0.91
2500–4999	1.09	0.27–4.45	0.91
≥5000	1.18	0.25–5.61	0.84

*p< 0.05.

^1^The variable unemployed includes student, housewife and volunteer.

^2^ Antillean Guilders, 1 ANG = 0.54 USA dollars and 0.47 EUR.

No significant interaction was observed between gender and age.

*Note*: Nagelkerke R^2^ = 0.175 (17.5%), Omnibus *(p = 0*.*00)*, and Hosmer and Lemeshow *(p = 0*.*62)* show a good fit of the model.

The qualitative data of our study support the results concerning the effect of age on the use of the internet, including social media use. An FGD conducted among the local youth (age range 19–24 years), e.g. showed that the younger generation received information regarding chikungunya mainly via social networks (Facebook). The younger participants of our study preferred to receive health information from social media because it offered them the opportunity to receive real-time information:

**Moderator:** *“Uhm*, *let us start*, *where did you get information about chikungunya*? *Where do you get it*?*”*

**Female 1:** *“Family*.*”*

**Moderator:** *“Family*.*”*

**Female 1:** *“From people who had chikungunya*.*”* **Female 2:** “I have received information from Facebook. On social media.” (laugh)

**Moderator:** *“You are used to it*.*”*

**Male 1:** *“I think*, *Facebook*.*”*

**Moderator:** *“Okay*. *You are on that thing all day*.*”*

(The youth was laughing)

**Moderator:** *“All right*.*”*

**Male 1:** *“Yes*, *it is easy*. *I do not want to wait until 20*:*00 for the news*. *The news circulates very fast on Facebook*.*”* **Male 2:** *“Yes*! *Social media”*. **Female 1:** *“On the other hand*, *many people were sharing their stories on Facebook*. *You know*, *tips*. *Thus*, *what to do and what not to do*.*”* **Male 1:** *“Yes*, *it belongs to social media*.*”*

**Moderator:** *“Is that true*? *That people post everything on Facebook*?*”*

**Female 2:** *“Yes*!*”* **Male 1:** *“To share their experiences*.*”* **Female 3:** *“Not their bodies*, *but their experiences*.*”*

FGD, The local youth (age range 19–24 years)

In general, the participants of both FGDs and IDIs indicated that information regarding dengue is shared by the local health authorities on a regular basis. Because of this, people have become familiar with the disease as well as with necessary preventive measures. However, when it comes to chikungunya, the participants of both FGDs and IDIs were not satisfied with the local health authorities. According to the majority of the participants, the official information regarding chikungunya coming from the local health authorities was late:

“People were criticising, ah, it went like a type of epidemic on the island. People had the idea that the minister did not act on time to stop the epidemic. I am not sure about the impact of the governmental programs, the programs regarding fumigation, on the community. However, people had the idea that the governmental actions were like too little too late (Dutch proverb: mustard after the meal), and it was on a small scale. Thus, the governmental actions were too late to prevent the epidemic.”

Female, teacher, age not documented, IDI.

The qualitative data analysis revealed that, as a result of the perceived delay in official information regarding chikungunya, people started to search for information. In order to verify and validate their opinions, they started to share their opinions regarding the transmission routes, symptoms, prevention and treatment options with people in their surroundings. Some participants reported turning to friends and family members for assistance in assigning meaning to the symptoms they were experiencing, and for advice on how to treat their condition. This could explain why many participants reported having received information regarding chikungunya via interpersonal channels.

### Risk perceptions toward dengue and chikungunya

A considerable proportion of the participants of this mixed-method study agreed that everyone in the community was potentially susceptible to DENV infection, regardless of his or her age. According to the participants of both FGDs and IDIs, DENV infection is not a severe disease, because the symptoms are flu-like. However, they were well-informed about the severe consequences of DENV infection. In the case of the survey participants, 67% and 60% of them fully agreed that DENV infection is a serious condition and that people who are infected could die of the infection, respectively. Based on these results, the majority of the participants had a moderate-high perceived susceptibility and a moderate-high perceived severity of DENV infection.

In the case of CHIKV infection, the majority of the participants of both FGDs and IDIs did not expect to be infected with CHIKV because they were not informed, before the epidemic, about a possible CHIKV infection outbreak. The qualitative data suggested that the perceived susceptibility and the perceived severity regarding CHIKV infection were attenuated due to the lack of RC. However, both were amplified when the television, radio, newspaper, and people started to share information regarding the high burden of chikungunya during the epidemic. When prompted to discuss gender and age differences related to CHIKV infection, a proportion of the participants of the FGDs and IDIs perceived that the youth was less at risk of CHIKV infection as their immune system was stated to be better than children, adults and older adults. However, the rest of the participants agreed that everyone in the community was susceptible to the condition, regardless of gender and age. The same shared perception was observed among the survey participants as almost half of the participants (47%) reported that not everyone in Curaçao ran a high risk of being infected with CHIKV. When participants were asked about the severity of CHIKV infection, all the participants of the qualitative study stated that it was a severe disease and 72% of the participants of the survey fully agreed with this statement. Based on these results, the majority of the participants had a moderate-low perceived susceptibility and moderate-high perceived severity regarding CHIKV infection.

### The influence of cultural schemas on information, perceptions and preferences

Information regarding the knowledge of the transmission route, preventive measures and treatment options of our study participants, was published previously [39, 44]. In this study, we explored which channels of information influenced the perceptions and preferences of the study participants. The results are presented here by the following key themes: the transmission route, preventive measures and treatment options.

### The transmission routes

Most of the participants of the FGDs and IDIs indicated that CHIKV and DENV are transmitted by infected mosquitoes. However, some of the participants reported that CHIKV was also transmitted via air, unhygienic conditions, water or contact with an infected individual. These results are in line with the published results of the quantitative survey [39]. From the participant’s narratives, we extracted the following shared culture-bound perceived etiologies: all viruses are the same, a virus is contagious, and something contagious is transmitted via contact with an infected person, air or water.

“I think it is a virus. I never thought that it was through a mosquito because I have been bitten several times by mosquitoes. It is a virus; It is like the flu (common cold).”

Female, housewife, age not documented, IDI.

The culture-bound perceived etiologies mentioned above were the basis for doubting the vector transmission route of DENV and CHIKV.

### Preventive measures

Various preventive measures were mentioned during the FGDs and IDIs. Participants suggested spraying insecticides in and outside the house, using plagatox (a mosquito incense usually made into a spiral with insecticidal dried powder or paste), larvicide *(e*.*g*. *Abate)* or repellent, the disposal of tires and bottles, removal of stagnant water breeding sites, cleaning of yards, and wearing long-sleeved clothing. Also, some participants indicated that eating healthy, using vitamins, and having a good immune system protects them against CHIKV and DENV infection:

**Female 1:** *“Hmm*, *yes*. *In my case*, *someone told me that I need to take precautionary measures*. *So*, *I started buying vitamins at that moment*. *So*, *I was using vitamins during my chikungunya infection*, *and I am still buying vitamins every month*, *in order to maintain a good immune system*. *So that I do not get that disease anymore*.*”* **Female 2:** *“Yes*, *so she thinks that eating healthy will protect her and you from being bitten*. *Right*?*”* **Female 1:** “*Yes*, *No*. *In my opinion*, *it is not about eating healthy; it is about vitamins*. *It is not about eating healthy*, *but the immune system needs to stay high so that it (chikungunya) does not come back*. *“***Female 2:** *“Yes*, *so that they do not get the disease anymore*.*”*

FGD 7, with survey interviewers.

According to the qualitative data, people thought that chikungunya was similar to a common cold so that they had the perception that healthy food and vitamins would improve or optimally maintain their immune system and subsequently also prevent them from acquiring CHIKV infection. These perceptions are based on the cultural schemas regarding preventive measures of the common cold. The most frequently recommended preventive measures reported in the FGDs and IDIs are presented in S7 Table. The data shown in this table highlights the diversity of information and perceptions that individuals have regarding preventive measures in the context of VBDs.

### Treatment options

The narratives of participants revealed that the individual’s shared cultural schemas regarding the treatment of diseases, *(e*.*g*. *the doctor knows how to cure diseases*, *herbs can cure diseases and fluid keeps ill people hydrated and reduces fever)* played an essential role in forming their perceptions and expectations regarding the treatment of, mainly, the CHIKV infection.

The participants reported that people went to the GP several times to seek medical care because the provided treatment, which was the use of painkillers *(e*.*g*. *paracetamol)*, was not providing satisfactory results. According to the qualitative data, people were not satisfied with the provided care of the GP, because the GP did not have a cure for CHIKV infection. Furthermore, some participants indicated that when they failed to find a cure for CHIKV infection, they became confused:

“Yes, because eh, I was angry, because eh, nobody was able to tell me what I needed to do. People were recommending different things; use corticosteroid injections, because you never know, maybe you can develop rheumatism in a later stage. There were too many insecurities.”

Female, translator, age not documented, IDI.

**Fig 1:**
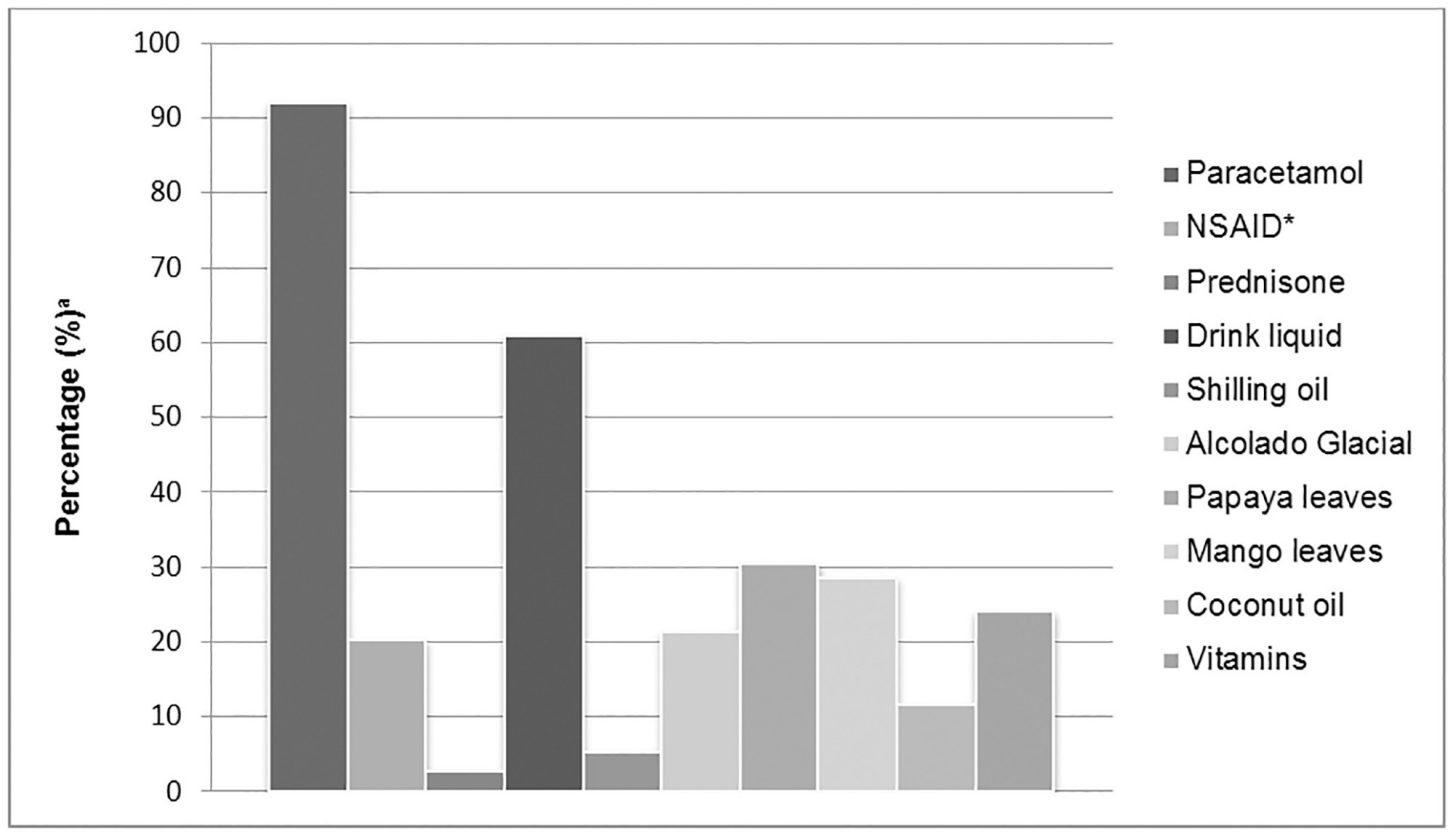
Treatment options for CHIKV infection. ^a^ Values are percentages of available participants who reported the remedies that they used to treat the CHIKV infection. Various options could be indicated. *The variable Non-Steroidal Anti-Inflammatory Drugs (NSAID) consists of the following medications ibuprofen, diclofenac and celecoxib. For one participant the data was missing on the following treatment strategies: paracetamol, drink liquid and Alcolado Glacial. This response was excluded from the data analysis (n = 338). For two participants there were missing data on the following treatment strategies: NSAID, coconut oil, mango leaves, shilling oil, vitamins, and Prednisone. These too were excluded from the data analysis (n = 337).

The qualitative data revealed that alternative medicine practitioners provided information regarding the use of papaya leaves to treat VBDs. The extract of papaya leaves was recommended because it was believed to increase the platelet levels in patients infected with DENV and CHIKV, and in turn, reduced the severity of the infection. Although the use of herbs was recommended by alternative medicine practitioners, it should be stated that herbs are a popular and traditional treatment option to treat diseases among people living on the island. The positive experiences of treating the common cold and other diseases with herbs had motivated infected individuals to use alternative medicine, such as papaya, mango and soursop leaves, to treat CHIKV and possibly DENV infection. The results of the survey showed that 30.5% of 339 participants reported having used papaya leaves, and 28.5% of 337 study participants used mango leaves to treat the CHIKV infection (Fig 1). The use of mango leaves was also recommended by alternative medicine practitioners, although the benefits are not documented. Also, a close analysis of the qualitative data revealed that the expectations regarding the results of the mentioned herbs were not met and this caused more confusion among the infected chikungunya patients:

“No, I heard about papaya leaves via my neighbour. She went back to the doctor after using papaya leaves because she wanted to try an injection. People were saying so many things, and when I asked: and did it work? They were still complaining. Weird, huh?”

Female, translator, age not documented, IDI.

Some participants of the FGDs and the IDIs had the perception that drinking plenty of liquids, mainly water, would help them combat the CHIKV infection. The survey showed that 61% of 338 participants reported drinking plenty of liquids to treat the mentioned disease. Drinking water or other types of refreshments were primarily used for keeping individuals who are sick hydrated and to reduce fever. Also, people have the perception that water helps cleanse the body and subsequently removes waste, including disease, from the body.

Furthermore, the quantitative data showed that a proportion of participants used Shilling oil and Alcolado Glacial to treat the symptoms of CHIKV infection. Alcolado Glacial is a mentholated splash lotion that is produced in Curaçao, and the benefits of it have been passed on from generation to generation. People use it for many purposes, for example, to relieve pain and flu-related symptoms.

This mixed-method study revealed that people used multiple treatment options to treat CHIKV infection (Fig 1). According to the participant’s narratives, people used several treatment options as they were in a situation of insecurity due to the fact that there was no treatment to cure the CHIKV infection:

**Moderator:** *“Okay*, *Many people used different types of medication*. *Can you explain why people use so many different medications*?*”*

**Male 1:** *“When someone sees another person using a medication*, *he or she will ask for the details or the person using the medication will share the details*. *I mean*, *the person that is ill wants to get better*. *Thus*, *he or she will also try the medication*.*”* **Male 2**: *“Yes*, *powerlessness*.*”* **Female 1:** *“It becomes an act of powerlessness*. *Because if you get so much information*, *in the end*, *you will not know what the truth is*, *and you will try what you hear*. *I am a person that can say no*. *I say*, *no*! *I am not drinking everything because with those things (alternative medicine) you have to be careful*, *because it does not work every time*. *You have to follow your feelings*. *When it (feelings) says okay you can try it*, *go ahead*! *However*, *do not drink everything that people recommend because you do not know what it does to you*. *It can be poisonous and make you sicker*.*”* **Male 1:** *“Good*.*”*

FGD 3, Souax

Noticeably, people were desperately searching for an effective cure. Fig 1 shows the wide range of interventions people were exploring to treat CHIKV infection.

### Trust in the channel of information

Some participants of the qualitative study opinionated that the government did not have a plan to inform the community and combat the epidemic, there was not enough workforce, and the government had other priorities at that specific point in time. Furthermore, some participants doubted the information provided by their GP. These disappointments with the government and the GPs could be linked to reduced trust in RC.

**Male 1:** *“The information regarding the vector of chikungunya was lacking*. *People were not aware of the possible actions that they could take*. *I think that chikungunya is a virus*, *and there is no medication against viruses*. *The consequence is that you cannot walk and as what (female name X) said*, *you need to rest*. *If someone says that he or she has medication for the virus*, *the person needs to know the effect of it*. *I heard that people tried everything that was recommended by the doctors*. *Painkillers were prescribed most of the time*. *This is clear*, *it is not a medication*, *but it helps to reduce the symptoms*. *The consequence was that people got confused because they thought that they got medication to cure the disease*, *while you as a health professional knew that there is no cure*.*”* **Male2:** *“Painkillers*.*”* **Male 1:**” Yes, *that does not help*. *I do not believe that it helps*.*”*

FGD 3, Souax

Both qualitative and quantitative data reveal that interpersonal channels of information played an essential role in the provision of information regarding DENV and CHIKV infection. As mentioned above, some participants reported turning to friends and family members for assistance in understanding their condition. This action could indicate the trust that the participants have in information coming from their family and friends. Some participants reported taking actions that family or friends were taking:

“*My niece and her mother had it before me*. *So*, *I heard about chikungunya from them*. *Everyone had chikungunya*, *and my sister did not go to the doctor*, *although my niece did*. *That is why I did not go to the doctor anymore*.*”*

Female, accountant, age not documented, IDI.

Many participants had a firm belief in the efficacy of local herbs provided by alternative medicine practitioners. According to the qualitative data, the information regarding the benefits of herbs and the use of alternative medicine practitioners have been handed down from generation to generation. It has been stated by several participants of the FGDs and IDIs that positive experiences of using herbs as a treatment option had influenced how people trust the information coming from alternative medicine practitioners nowadays.

## Discussion

The main aim of this study was to understand RC regarding VBDs in the social context and from the audience’s point of view. We found that television, radio and newspapers were the three primary channels of information about dengue and chikungunya. However, interpersonal channels of information such as family, friends and neighbours also played an essential role in providing information regarding the mentioned VBDs. Our results are in line with those of others [50–52]. We also found that the use of internet to find information on VBDs varied by participant’s age and gender. The use of internet diminished with increasing age and women were more likely to use internet compared to men. These results are in line with the findings of the CBS of Curaçao (CBS, ICT & Media survey 2017) and with the literature [52, 53]. Our multivariate model, including age, gender, education, occupation and income, explains only 17.5% *(Nagelkerke R*^*2*^ = *0*.*175)* of internet usage. Using the internet for information on chikungunya may be less influenced by common socio-demographic characteristics and may be better explained by other factors such as personality traits *(e*.*g*. *extraversion)* [54].

The qualitative data of our study showed how the risk perception regarding CHIKV infection was attenuated due to the lack of RC before the epidemic and how the risk perception was amplified by the increased exposure of information, as well as the direct and indirect experience of the physical manifestation of CHIKV infection in the peak of the chikungunya epidemic. Based on the participant’s narratives, we believe that the media including television, radio, newspapers and interpersonal channels of information played an essential role in the amplification of the risk of individuals, especially in the context of chikungunya. The findings of a recent study published by Chan et al. support our results [34]. Chan et al. showed that changes in the volume of information in media were followed by several changes in individuals risk perceptions and protective behaviour regarding the Zika virus in the United States [34]. They also found that social media coverage was positively correlated with changes in risk perception while television and newspapers were positively correlated with changes in protective behaviours. These findings revealed that the influence of media on risk perceptions and behaviour could vary by the volume of information and type of channel of information [34]. However, more research is needed to investigate these differences.

With regards to the risk perception of people, our results showed that the majority of the participants had a moderate-high perceived susceptibility and a moderate-high perceived severity of DENV infection. However, the qualitative data show that people considered it also to be “merely flu-like”. We believe this relatively high perceived disease severity of DENV infection is biased by the knowledge of the participants gained over the last years. It is possible that the participants are aware of the threat of DENV infection and report it, but that on the other hand, they do not really consider DENV infection as a threat as there have hardly been any reports of severe dengue disease on the island. This phenomenon could well be the reason for an initially reduced risk perception of the CHIKV infection. More research is needed to understand the feeling of threat with respect to VBDs among individuals living in Curaçao and to assess the role of other factors, e.g. heuristics, in this context.

Furthermore, we found that individual cultural schemas played an essential role in forming individuals’ perceptions regarding the preventive measures and treatment options of VBDs, especially in the case of chikungunya. The information regarding the influence of cultural schemas on the perceptions and behaviour of individuals in the context of VBDs is lacking. A recent paper published by Metta et al. showed that cultural schemas played a role in malaria self-care among adults in Tanzania, especially regarding the use of herbs in healing multiple conditions [26]. A similar cultural schema was observed in our study, with regard to using herbs to treat CHIKV and possible DENV infection. We showed that individuals’ cultural schemas are possible cues to action, which instigated the health-seeking behaviour of individuals. Also, the results of our study showed that people got confused when their reality *(e*.*g*. *herbs did not cure or reduce the symptoms of the CHIKV infection)* did not fit in their cultural schema *(e*.*g*. *herbs heal diseases)*. RC experts will need to include aspects of cultural *schemas (e*.*g*. *the use of herbs in the treatment of disease)* in their RC strategies.

Lastly, the data of this study also highlighted trust issues between the community and the local health authorities, including the general practitioners. As the participants perceived that information regarding CHIKV infection came when the community was already experiencing the condition, they expressed disappointment and showed signs of reduced trust in the health authorities. There was less trust in the government because people perceived that the measures performed by the government were not enough to protect them against health insecurities; in this case, the CHIKV infection epidemic. This implies that governmental actions during this epidemic would have influenced people’s trust in the government. There was less trust in general practitioners because the patients doubted the efficacy of the treatments. We believe that the power relations between health practitioners and patients are changing in Curaҫao. Health practitioners can no longer assume that patients will trust them because of their power/position in society. Trust has been found to play an essential role in predicting risk perceptions and behaviours of individuals [55]. In the case of distrust, people will doubt the information that the channel is providing and this in turn will affect not only their risk perception and their behaviour but also RC efforts as such in a negative way. In other words, negative trust issues should be addressed in RC.

### Limitations and strengths

The current study was limited by its cross-sectional retrospective design and is susceptible to recall bias. The study participants consisted of more females than males; this might limit the generalisability of our results. However, this might not be a significant issue, because women are mainly responsible for the housekeeping, family care and the provision of information to the family [56, 57]. The wide confidence intervals that were found for all socio-demographic characteristics may be explained by the small number of participants in each category. Therefore, “age” was used as a continuous variable in the regression model. A strength of the study was its mixed-method design. Also, our study design offered the opportunity to understand RC from the audience’s point of view, which could help RC experts to understand their target population comprehensively. Even though our sample size is small, our results on internet use correlate with the results of the CBS which was based on a representative sample of the population.

### Conclusions and recommendations

The findings of this study form an important contribution to the research field of RC, because to our knowledge, no studies combining qualitative and quantitive research methods based on the SARF, HBM and cultural schemas theory to understand RC from the audience’s perspective have been published to date. We found that there are different factors *(e*.*g*. *1*. *the timing of sharing information*, *2*. *the channel of information*, *3*. *the volume of information*, *4*. *trust in the source of information*, *5*. *previous experience with a disease*, *and 6*. *cultural schemas)* that influence how people deal with risk. Health authorities should target these factors in future RC strategies. We recommend broadening the use and scope of media platforms to share information on a timely basis and with the content that convinces people of the seriousness of the matter. The health authorities can customise their channels of information by using more social media platforms to reach the younger generation and other groups in the community. We recommend to evaluate the manner in which social media was used and to what extent people were aware of the existence of these platforms to achieve the desired impact. As the health authorities are mistrusted by the public, more efforts need to be made to provide accurate information to the community through a transparent process. Lastly, further research should take more individual characteristics such as cultural schemas and heuristics into account when studying the influence of RC on risk perception and behaviour of individuals.

## Supporting information

S1 Table Characteristics of the study participants of the FGDs and IDIs. Adapted from: Elsinga J, van der Veen HT, Gerstenbluth I, Burgerhof JGM, Dijkstra A, Grobusch MP, et al. Community participation in mosquito breeding site control: an interdisciplinary mixed methods study in Curacao. Parasit Vectors. 2017;10(1):434.
(DOCX)

S2 Table Coding list for FGDs and IDIs. (DOCX)

S3 Table Socio-demographic characteristics of the survey participants. ^a^ Total is 338. ^b^ Total is 332. ^c^ Antillean Guilders, 1 ANG = 0.54 USA dollars and 0.47 EUR. Adapted from: Elsinga J, van der Veen HT, Gerstenbluth I, Burgerhof JGM, Dijkstra A, Grobusch MP, et al. Community participation in mosquito breeding site control: an interdisciplinary mixed methods study in Curacao. Parasit Vectors. 2017;10(1):434.
(DOCX)

S4 Table Selected comparisons between socio-demographic characteristics and the use of social media and the internet to seek information regarding chikungunya. ^1^Antillean Guilders, 1 ANG = 0.54 USA dollars and 0.47 EUR. *Fisher-Freeman-Halton exact test. ^ Chi-square test.
(DOCX)

S5 Table Univariate analysis of socio-demographic characteristics associated with the use of social media to seek information regarding chikungunya. ^1^The variable unemployed includes student, housewife and volunteer. ^2^Antillean Guilders, 1 ANG = 0.54 USA dollars and 0.47 EUR. ^3^For two participants the data regarding social media usage was missing. These two participants were excluded from the data analysis (n = 337). ^4^For eight participants the data regarding social media usage was missing. These eight participants were excluded from the data analysis (n = 331). *Significance was determined at an alpha level of 0.10.
(DOCX)

S6 Table Multivariate analysis of socio-demographic characteristics associated with the use of social media to seek information regarding chikungunya. ^1^The variable unemployed includes student, housewife and volunteer. ^2^Antillean Guilders, 1 ANG = 0.54 USA dollars and 0.47 EUR. *Significance was determined at an alpha level of 0.05. *Note*: Nagelkerke R^2^ = 0.181 (18.1%), Omnibus *(p = 0*.*00)*, and Hosmer and Lemeshow *(p = 0*.*31)* show a good fit of the model.
(DOCX)

S7 Table Preventive measures reported in the FGDs and IDIs. (DOCX)

S1 Checklist STROBE statement—Checklist of items that should be included in reports of observational studies. (DOCX)
