# Supplementary material for: Understanding risk communication for prevention and control of vector-borne diseases: A mixed-method study in Curaçao
Source: PLoS Negl Trop Dis. 2020 Apr 13;14(4):e0008136. doi: 10.1371/journal.pntd.0008136 (PMC7153856; doi:10.1371/journal.pntd.0008136)
Supplement: S1 Table — Adapted from: Elsinga J, van der Veen HT, Gerstenbluth I, Burgerhof JGM, Dijkstra A, Grobusch MP, et al. Community participation in mosquito breeding site control: an interdisciplinary mixed methods study in Curacao. Parasit Vectors. 2017;10(1):434. (DOCX) [file pntd.0008136.s001.docx]

**S1 Table.**Characteristics of the study participants of the FGDs and IDIs

|  | **# participants** | **# Female** | **Age range** |
| --- | --- | --- | --- |
| **FGDs (n=7)** |  |  |  |
| Residents from the Netherlands | 8 | 6 | 61-71 |
| Local youth | 4 | 2 | 19-24 |
| Koraalspecht | 10 | 10 | 55-97 |
| Seru Fortuna | 9 | 8 | 18-70 |
| Rooi Santu | 8 | 4 | 51-80 |
| Souax | 7 | 4 | 34-72 |
| Interviewers of the survey | 4 | 3 | 64-67 |
|  |  |  |  |
| **IDIs (n=20)** |  |  |  |
| Participants infected with the CHIKV | 20 | 12 | 36-87 |
| Family members | 5 | 2 |  |

Adapted from: Elsinga J, van der Veen HT, Gerstenbluth I, Burgerhof JGM, Dijkstra A, Grobusch MP, et al. Community participation in mosquito breeding site control: an interdisciplinary mixed methods study in Curacao. Parasit Vectors. 2017;10(1):434.
