# Supplementary material for: Understanding risk communication for prevention and control of vector-borne diseases: A mixed-method study in Curaçao
Source: PLoS Negl Trop Dis. 2020 Apr 13;14(4):e0008136. doi: 10.1371/journal.pntd.0008136 (PMC7153856; doi:10.1371/journal.pntd.0008136)
Supplement: S2 Table — (DOCX) [file pntd.0008136.s002.docx]

**S2 Table.** Coding list for FGDs and IDIs

| **Family codes** | **Codes** |
| --- | --- |
| Channel of information  Preferred channels of information | Television  Newspaper  Radio  Family  Friends  Colleagues  Conventional medicine practitioners  Alternative medicine practitioners  Flyers  Social media  Internet *(e.g. websites, google)*  International media  Schools  Community centres  Government *(e.g. minister)*  Public Health Department (G&Gz previously called GGD) |
| The user of the channel of information | Younger generation  The older generation |
| Risk perception | Susceptibility of dengue virus infection  The severity of dengue virus infection  Susceptibility of chikungunya virus infection  The severity of chikungunya virus infection  The feeling of fear |
| Transmission routes | Water  Unhygienic conditions  Air  Bite of an infected mosquitoes  Contact with an infected individual |
| Symptoms | Headache  Muscle pain  Joint swelling  Rash |
| Preventive measures | Spraying insecticides  Plagatox  Larvicide *(e.g. Abate)*  Repellent  The disposal of tires and bottles  Removal of stagnant water breeding sites  Cleaning of yards  Wearing long-sleeved clothing  Nets  Eating healthy  Vitamins  Good immune system |
| Treatment options | Painkillers *(e.g. paracetamol)*  Drink liquid  Alcolado Glacial  Vitamines  Coconut oil  Mango leaves  Papaya leaves  Shilling oil  Prednisone |
| Trust in the channel of information | Sources trusted  Source not trusted  Negative  Positive  Information was late  Unclear information |
