# Supplementary material for: Understanding risk communication for prevention and control of vector-borne diseases: A mixed-method study in Curaçao
Source: PLoS Negl Trop Dis. 2020 Apr 13;14(4):e0008136. doi: 10.1371/journal.pntd.0008136 (PMC7153856; doi:10.1371/journal.pntd.0008136)
Supplement: S3 Table — a Total is 338. b Total is 332. c Antillean Guilders, 1 ANG = 0.54 USA dollars and 0.47 EUR. Adapted from: Elsinga J, van der Veen HT, Gerstenbluth I, Burgerhof JGM, Dijkstra A, Grobusch MP, et al. Community participation in mosquito breeding site control: an interdisciplinary mixed methods study in Curacao. Parasit Vectors. 2017;10(1):434. (DOCX) [file pntd.0008136.s003.docx]

**S3 Table.** Socio-demographic characteristics of the survey participants

|  | **Total (N=339)** | **N (%)** |
| --- | --- | --- |
| **Age** | | |
| 18-50 | 164 | 48.4 |
| *≥*51 | 175 | 51.6 |
| **Gender** | | |
| Female | 247 | 72.9 |
| Male | 92 | 27.1 |
| **Education** | | |
| Illiterate and primary school | 80 | 23.6 |
| Secondary school | 128 | 37.8 |
| Intermediate vocational school | 84 | 24.8 |
| Higher vocational education | 47 | 13.9 |
| **Occupation ^a^** | | |
| Unemployed/student/housewife/ volunteer | 63 | 18.6 |
| Paid job (manual) | 144 | 42.6 |
| Paid job (not manual) | 67 | 19.8 |
| Retired | 64 | 18.9 |
| **Income ^b,c,^** | | |
| 0-999 ANG/ month | 35 | 10.5 |
| 1000-2499 ANG/ month | 136 | 41.0 |
| 2500-4999 ANG/ month | 118 | 35.5 |
| ≥5000 ANG/ month | 43 | 13.0 |

^a^ Total is 338

^b^ Total is 332

^c^ Antillean Guilders, 1 ANG= 0.54 USA dollars and 0.47 EUR

Adapted from: Elsinga J, van der Veen HT, Gerstenbluth I, Burgerhof JGM, Dijkstra A, Grobusch MP, et al. Community participation in mosquito breeding site control: an interdisciplinary mixed methods study in Curacao. Parasit Vectors. 2017;10(1):434.
