# Supplementary material for: Understanding risk communication for prevention and control of vector-borne diseases: A mixed-method study in Curaçao
Source: PLoS Negl Trop Dis. 2020 Apr 13;14(4):e0008136. doi: 10.1371/journal.pntd.0008136 (PMC7153856; doi:10.1371/journal.pntd.0008136)
Supplement: S4 Table — 1Antillean Guilders, 1 ANG = 0.54 USA dollars and 0.47 EUR. *Fisher-Freeman-Halton exact test. ^ Chi-square test. (DOCX) [file pntd.0008136.s004.docx]

**S4 Table.** Selected comparisons between socio-demographic characteristics and the use of social media and the internet to seek information regarding chikungunya

| **Age vs Social media** | **Users n (%)** | **Total number of subjects** | ***p-*value** |
| --- | --- | --- | --- |
| 18-50 years  ≥ 51 years | 30 (18.4)  10 (5.7) | 163  174 | < 0.001^^^ |
| **Age vs Internet** | **Users n (%)** | **Total number of subjects** | ***p-*value** |
| 18-50 years  ≥ 51 years | 40 (24.5)  12 (6.9) | 163  174 | < 0.001^^^ |
| **Gender vs Social media** | **Users n (%)** | **Total number of subjects** | ***p-*value** |
| Male  Female | 6 (6.6)  34 (13.8) | 91  246 | 0.07^^^ |
| **Gender vs Internet** | **Users n (%)** | **Total number of subjects** | ***p-*value** |
| Male  Female | 7 (7.7)  45 (18.3) | 91  246 | 0.02^^^ |
| **Education vs Social media** | **Users n (%)** | **Total number of subjects** | ***p-*value** |
| Illiterate and primary school  Secondary school  Intermediate vocational school  Higher vocational education | 4 (5.0)  19 (15.0)  8 (9.6)  9 (19.1) | 80  127  83  47 | 0.05^^^ |
| **Education vs Internet** | **Users n (%)** | **Total number of subjects** | ***p-*value** |
| Illiterate and primary school  Secondary school  Intermediate vocational school  Higher vocational education | 4 (5.0)  21 (16.5)  15 (18.1)  12 (25.5) | 80  127  83  47 | 0.01^^^ |
| **Occupation vs Social media** | **Users n (%)** | **Total number of subjects** | ***p-*value** |
| Unemployed  Paid job (manual)  Paid job (not manual)  Retired | 4 (6.3)  17 (11.9)  17 (25.4)  2 (3.1) | 63  143  67  64 | < 0.001^^^ |
| **Occupation vs Internet** | **Users n (%)** | **Total number of subjects** | ***p-*value** |
| Unemployed  Paid job (manual)  Paid job (not manual)  Retired | 7 (11.1)  25 (17.5)  17 (25.4)  3 (4.7) | 63  143  67  64 | < 0.01^^^ |
| **Income** (ANG/*month)^1^* **vs Social media** | **Users n (%)** | **Total number of subjects** | ***p-*value** |
| 0 - 999  1000 – 2499  2500 – 4999  ≥5000 | 3 (8.6)  10 (7.4)  16 (13.6)  9 (20.9) | 35  135  118  43 | 0.09^*^ |
| **Income** (ANG/*month)^1^* **vs Internet** | **Users n (%)** | **Total number of subjects** | ***p-*value** |
| 0 – 999  1000 – 2499  2500 – 4999  ≥5000 | 3 (8.6)  16 (11.9)  21 (17.8)  10 (23.3) | 35  135  118  43 | 0.16^^^ |

^1^Antillean Guilders, 1 ANG= 0.54 USA dollars and 0.47 EUR

*Fisher-Freeman-Halton exact test, ^ Chi-square test
