# Supplementary material for: Understanding risk communication for prevention and control of vector-borne diseases: A mixed-method study in Curaçao
Source: PLoS Negl Trop Dis. 2020 Apr 13;14(4):e0008136. doi: 10.1371/journal.pntd.0008136 (PMC7153856; doi:10.1371/journal.pntd.0008136)
Supplement: S5 Table — 1The variable unemployed includes student, housewife and volunteer. 2Antillean Guilders, 1 ANG = 0.54 USA dollars and 0.47 EUR. 3For two participants the data regarding social media usage was missing. These two participants were excluded from the data analysis (n = 337). 4For eight participants the data regarding social media usage was missing. These eight participants were excluded from the data analysis (n = 331). *Significance was determined at an alpha level of 0.10. (DOCX) [file pntd.0008136.s005.docx]

**S5 Table.** Univariate analysis of socio-demographic characteristics associated with the use of social media to seek information regarding chikungunya

|  |  |  | **Use of social media** | |  |
| --- | --- | --- | --- | --- | --- |
| **Variables** | **Users**  **n (%)** | **Total number of subjects** | **Crude OR 95% CI for exp b** | | ***p-*value** |
| **Age**  *(years)*^3^ | 40 (11.9) | 337 | 0.95 | 0.93 – 0.98 | 0.00* |
| **Gender**^3^  Male  Female | 6 (6.6)  34 (13.8) | 91  246 | 1  2.27 | -  0.92 – 5.61 | -  0.01* |
| **Education**^3^  Illiterate and primary school  Secondary school  Intermediate vocational school  Higher vocational education | 4 (5.0)  19 (15.0)  8 (9.6)  9 (19.1) | 80  127  83  47 | 1  3.34  2.03  4.50 | -  1.09 – 10.22  0.58 – 7.02  1.30 – 15.56 | 0.07*  0.03*  0.26  0.02* |
| **Occupation**^1,3^  Unemployed  Paid job (manual)  Paid job (not manual)  Retired | 4 (6.3)  17 (11.9)  17 (25.4)  2 (3.1) | 63  143  67  64 | 1  1.99  5.01  0.48 | -  0.64 – 6.17  1.58 – 15.88  0.08 – 2.70 | < 0.001*  0.23  0.01*  0.40 |
| **Income** *(ANG/month)^2,4^*  0 - 999  1000 – 2499  2500 – 4999  ≥5000 | 3 (8.6)  10 (7.4)  16 (13.6)  9 (20.9) | 35  135  118  43 | 1  0.85  1.67  2.82 | -  0.22 – 3.28  0.46 – 6.11  0.70 – 11.37 | 0.09*    0.82  0.44  0.14 |

^1^The variable unemployed includes student, housewife and volunteer

^2^Antillean Guilders, 1 ANG= 0.54 USA dollars and 0.47 EUR

^3^For two participants the data regarding social media usage was missing. These two participants were excluded from the data analysis (n=337).

^4^For eight participants the data regarding social media usage was missing. These eight participants were excluded from the data analysis (n=331).

*Significance was determined at an alpha level of 0.10.
