# Supplementary material for: Understanding risk communication for prevention and control of vector-borne diseases: A mixed-method study in Curaçao
Source: PLoS Negl Trop Dis. 2020 Apr 13;14(4):e0008136. doi: 10.1371/journal.pntd.0008136 (PMC7153856; doi:10.1371/journal.pntd.0008136)
Supplement: S6 Table — 1The variable unemployed includes student, housewife and volunteer. 2Antillean Guilders, 1 ANG = 0.54 USA dollars and 0.47 EUR. *Significance was determined at an alpha level of 0.05. Note: Nagelkerke R2 = 0.181 (18.1%), Omnibus (p = 0.00), and Hosmer and Lemeshow (p = 0.31) show a good fit of the model. (DOCX) [file pntd.0008136.s006.docx]

**S6 Table.** Multivariate analysis of socio-demographic characteristics associated with the use of social media to seek information regarding chikungunya

| **Use of social media** | | | | |  |
| --- | --- | --- | --- | --- | --- |
| **Variables** | | **OR** | | **95% CI for exp b** | ***p*-value*** |
| **Age** *(years)* | 0.96 | | 0.93 – 0.99 | | 0.02* |
| **Gender** *(Female)*  **Education**  Illiterate and primary school  Secondary school  Intermediate vocational school  Higher vocational education  **Occupation**^1^  Unemployed  Paid job (manual)  Paid job (not manual)  Retired  **Income** *(ANG/month)^2^*  0 - 999  1000 – 2499  2500 – 4999  *≥*5000 | 2.54  1  1.33  0.66  0.83  1  2.68  4.82  1.81  1  0.59  0.89  1.15 | | 0.93 – 6.97  -  0.38 – 4.67  0.16 – 2.80  0.18 – 3.84  -  0.72 – 9.98  1.17 – 19.85  0.23 – 14.57  -  0.14 – 2.49  0.21 – 3.84  0.23 – 5.83 | | 0.07  0.52  -  0.65  0.58  0.82  0.16  -  0.14  0.03*  0.57  0.66  -  0.47  0.87  0.86 |

^1^The variable unemployed includes student, housewife and volunteer

^2^Antillean Guilders, 1 ANG= 0.54 USA dollars and 0.47 EUR

*Significance was determined at an alpha level of 0.05.

*Note:* Nagelkerke R^2^=0.181 (18.1%), Omnibus *(p=0.00),* and Hosmer and Lemeshow *(p=0.31)* show a good fit of the model.
