# Supplementary material for: Understanding risk communication for prevention and control of vector-borne diseases: A mixed-method study in Curaçao
Source: PLoS Negl Trop Dis. 2020 Apr 13;14(4):e0008136. doi: 10.1371/journal.pntd.0008136 (PMC7153856; doi:10.1371/journal.pntd.0008136)
Supplement: S7 Table — (DOCX) [file pntd.0008136.s007.docx]

**S7 Table.** Preventive measures reported in the FGDs and IDIs

| Coils *(a type of insecticide)* | *“what is it again? That spiral. What is the name again? Plagatox. Plagatox.”*  Female, accountant, age not documented, IDI. |
| --- | --- |
| Larvicide | *“Yes, you can do it yourself, because you can get Abate here.”*  Male, retired, 68 years, IDI. |
| Environmental management | “ Making sure that there is no stagnant water in the area.”  Female, art historian and History teacher, age not documented, IDI. |
| Insecticide | “Aerosol.”  Male, Loader Operator, age not documented, IDI. |
| Environmental management | “Eliminate empty pots, tires or bottles from your yard.”  Male, retired, 68 years, IDI. |
| Clothing that minimises skin exposure and repellent | *“Prevention? Yes eh, wrap yourself up.. eh? Space clothing, yes space clothing. Anyway use long sleeves, and use eh.. that thing against* *insects.”*  Male, general practitioner, age not documented, IDI. |
| Vitamins and herbs | Interviewer: *“Okay, how can people protect themselves against chikungunya?”*  Female 1: *“Drink herbs and eh… Vitamin C.”*  FGD 5, Koraal Specht |
| Healthy food | Interviewer: *“But what do you think that people could do to protect themselves against chikungunya?*  Male 1: *“To protect themselves?”*  Interviewer: *“Hmm.”*  Male 1: *“Do not leave anything in your garden that can hold water. Plants and things like that, look good! Also, they (people) need to eat well in order to maintain a stable resistance.”*  FGD 3, Souax |
